# Patients Hospitalized for Ischemic Stroke and Intracerebral Hemorrhage in France: Time Trends (2008–2019), In-Hospital Outcomes, Age and Sex Differences

**DOI:** 10.3390/jcm11061669

**Published:** 2022-03-17

**Authors:** Valérie Olié, Clémence Grave, Philippe Tuppin, Gauthier Duloquin, Yannick Béjot, Amélie Gabet

**Affiliations:** 1 Department of Non-Communicable Diseases, Santé Publique France, 94410 Saint-Maurice, France; valerie.olie@santepubliquefrance.fr (V.O.); clemence.grave@santepubliquefrance.fr (C.G.); 2 Department of Health Studies and Statistics, Caisse Nationale de l’Assurance Maladie, 75020 Paris, France; philippe.tuppin@assurance-maladie.fr; 3 Dijon Stroke Registry, University Hospital of Burgundy, 21000 Dijon, France; gauthier.duloquin@chu-dijon.fr (G.D.); yannick.bejot@chu-dijon.fr (Y.B.)

**Keywords:** ischemic stroke, intracerebral hemorrhage, hospitalization, outcomes, temporal trends

## Abstract

Background: Rates of patients hospitalized for stroke increased among people aged under 65 years in France, as has been found in other countries. Methods: To analyze time trends in the rates of patients hospitalized for ischemic stroke (IS) and intracerebral hemorrhage (ICH) in France between 2008 and 2019 and determine related short-term outcomes mainly, we selected all patients hospitalized for stroke using the French national hospital database. Results: The average annual percentage change in the rates of patients hospitalized for IS increased significantly in men and women aged 50–64 years (+2.0%) and in men aged 18–34 years (+1.5%) and 35–44 years (+2.2%). A decrease in the average annual percentage change was observed for IS among people aged over 75 years and among those over 50 years for ICH. After adjustment on confounding factors, women were less likely to die in hospital. Case fatality rates decreased overtime in all age groups for both sexes, with a more pronounced decrease for IS than ICH. Conclusions: The increasing trend of IS among adults under 65 years is ongoing, highlighting the urgent need for stroke prevention programs in that age. For the first time, we recorded a decrease in the rates of patients hospitalized for ICH among the population over 50 years.

## 1. Introduction

Stroke is one of the main causes of adult disability and death in both men and women [1]. Despite the overall declines in age-standardized stroke incidence, death, and disability-adjusted life year (DALY) rates, this trend has noticeably slowed down over the past decade 2010–2019 compared with the previous decade 2000–2009, while the global age-standardized prevalence of stroke remained stable in high-income countries from 2010 to 2019 [1,2]. According to the Global Burden of Disease Study, a significant increase in stroke prevalence and incidence rates in terms of ischemic stroke (IS) was found in both men and women aged under 70 years between 1990 and 2019, with even faster increases observed from 2010 to 2019 [1]. A stable or increasing trend in stroke incidence among middle-aged people was recently observed in the USA, Canada, Australia, and European countries including France [3,4,5,6,7,8,9,10]. Several studies have predicted a substantial increase in the number of stroke cases in high-income countries by 2030 and 2050, associated with almost a doubling of healthcare costs related to stroke by then [1,11,12,13], as supported by projections from the Dijon Stroke Registry for France [14]. 

The aim of this study was to analyze trends in the rates of patients hospitalized for IS and intracerebral hemorrhage (ICH) in France between 2008 and 2019 according to sex and age group. We also investigated sex differences regarding admissions to acute stroke care units and in-hospital case fatality rates in the most recent period (2018–2019).

## 2. Methods

### 2.1. Data Sources

We used data from the French national hospital database, which collects all hospital stays in France from both public and private hospitals, covering a population of around 66 million inhabitants [15]. This database is linked to the French national healthcare database (SNDS) [15]. Data were extracted from 2008 to 2019 to avoid potential bias due to the COVID-19 pandemic in 2020 and 2021. 

### 2.2. Stroke Definition

Hospitalized IS and ICH cases were identified using the International Classification of Diseases, 10th revision (ICD-10). IS and ICH were recorded as the main diagnosis of the stay in a hospital or specialty care unit. IS was defined as codes I63 and I64, where I64 refers to undetermined stroke (i.e., ischemic strokes from undetermined etiology; this code is used less and less and represented only 5% of IS in 2019), whereas ICH was distinguished using code I61. Patients with a G46 code were also considered to have an IS if a code for IS was also mentioned as an associated diagnosis.

### 2.3. Population Selection

For each year between 2008 and 2019, we selected all patients with at least one hospitalization for stroke in France. For each patient, only the first stroke event was included. The first stroke event was defined using the first hospital stay for stroke.

### 2.4. Data Collection

The following sociodemographic characteristics were collected: age, sex, and department (that is, administrative division) of residence. During the hospital stay for stroke, we recorded medical and management information: comorbidities reported during hospitalization as defined by the Charlson index score [16], hemiplegia or paraplegia, aphasia, admission to an acute stroke care unit, admission to an intensive care unit (including resuscitation unit), admission to a geriatric unit, thrombolysis, mechanical thrombectomy (MT), length of overall hospital stay, and in-hospital death. MT was not recorded in healthcare databases until August 2017. Acute stroke care units have been introduced into French hospitals since 2003. In 2007, only 33 units were recorded. Since this date, the opening of acute stroke care units has continued to progress in France, which counted 147 such units in 2019.

### 2.5. Statistical Analyses

Annual rates of patients hospitalized for stroke were computed globally by sex, age group, and type of stroke (IS or ICH) using the national census population data provided by the National Institute of Statistics and Economic Studies. Rates were standardized based on the age structure of the 2010 European population. Temporal trends were studied using Poisson regression models while taking into account overdispersion. Average annual percentage changes were estimated for the rates and numbers of patients hospitalized for stroke according to sex, age group, and type of stroke. The in-hospital case fatality rate was defined by dividing the number of patients who died in hospital by the overall number of hospitalized patients. The association between sex and non-admission to an acute stroke care unit was studied for the most recent years (2018–2019) using multivariate logistic regression. The model was adjusted for age, admission to a geriatric unit during the hospital stay, Charlson index score (categorized in five class), presence of aphasia, hemiplegia, or paraplegia, and the number of beds available in acute stroke care units in the department where the patient lives. This analysis was restricted to the patients living in a department with at least one hospital equipped with an acute stroke care unit. A similar method was used to analyze the association between sex and in-hospital case fatality rate. Finally, the incidence rate ratio (IRR) comparing the rate of patients hospitalized for stroke between women and men was computed using Poisson regression. Statistical analyses were conducted using SAS Enterprise Guide 9.4.6.0 (SAS Inc., Cary, NC, USA). 

### 2.6. Ethical Approval and Data Availability

In line with the French governmental regulations and the National Ethics Committee, no patient consent was required. The databases used in the study contained pseudonymized patient information. Furthermore, full access to the SNDS, which includes the French national hospital database, is granted to the National Agency for Public Health (Santé Publique France) by French law (Code de la Santé Publique: articles L. 1461-3 I 2 and R. 1461-12) and decree (regulatory decision DE-2011-078).

## 3. Results

### 3.1. Epidemiology of Hospitalized Stroke Patients

**Figure 1:**
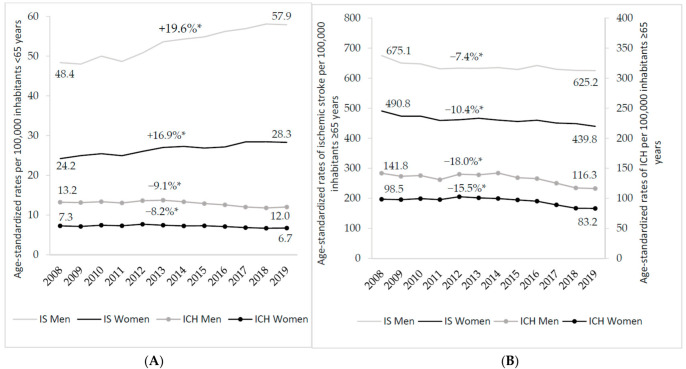
Time trends in age-standardized ^†^ rates of patients hospitalized for ischemic stroke (IS) and intracerebral hemorrhage (ICH) between 2008 and 2019 in France according to sex and age group. (**A**) Among inhabitants under 65 years. (**B**) Among inhabitants over 65 years. Gray: men; black: women. * *p*-value for trend < 0.05. ^†^ Rates standardized on the age structure of the 2010 European population census.

In 2019, 97,319 patients were hospitalized for IS and 18,722 for ICH (Table 1). The corresponding age-standardized rates per 100,000 inhabitants were 136.2 for IS and 26.5 for ICH, with higher rates in men than in women (Table 1). Age-standardized rates of IS remained stable in men between 2008 and 2019 (Supplemental Figure S1), whereas a significant decrease was observed in women (from 115.2 in 2008 to 108.5 in 2019 per 100,000; Supplemental Figure S1). Among inhabitants aged under 65 years, IS rates increased in both men (from 48.4 to 57.9 per 100,000) and women (from 24.2 to 28.3), whereas ICH rates remained stable in both men and women (Figure 1A). Among inhabitants aged over 65 years, age-standardized rates decreased in both men and women for all types of strokes, with a larger decrease in ICH recorded since 2013–2014 (Figure 1B). Although age-standardized rates were generally higher in men compared with women, the sex IRR (women to men) presented in Supplemental Figure S2 highlighted several changes with age. Women had higher rates of IS at the age of 18–34 years. Furthermore, women progressively had more similar age-standardized rates of both IS and ICH compared with men with increasing age above 75 years (Supplemental Figure S2). 

**Figure 2:**
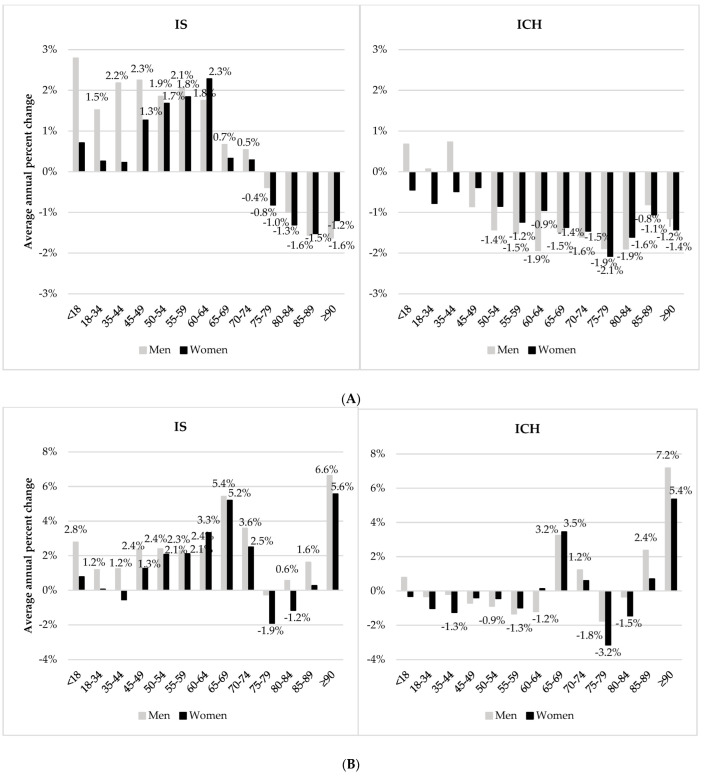
Average annual time trends ^†^ in rates and numbers of patients hospitalized for ischemic stroke (IS) and intracerebral hemorrhage (ICH) between 2008 and 2019 in France according to sex and age group. (**A**) Average annual percentage change in the rates of patients hospitalized for stroke. (**B**) Average annual percentage change in the absolute number of patients hospitalized for stroke. ^†^ Only significant average annual percentage changes are reported in the figure.

In terms of the average annual percentage change in the rates of patients hospitalized for IS by 5-year age groups, we recorded similar significant increases for both men and women aged 50–64 years, with a significant increase in men aged 18–34 and 35–44 years (Figure 2A). Regarding the rates of patients hospitalized for ICH, the average annual percentage change fell among people aged over 50 years. The corresponding average annual change in the absolute number of patients increased, which was even greater for IS in people aged 45–74 years (Figure 2B). A substantial increase in both IS and ICH was observed in men and women aged over 90 years. Overall, the absolute number of IS cases increased significantly in both men and women, unlike the absolute number of ICH cases, which remained stable in both men and women.

### 3.2. Acute Care and In-Hospital Case Fatality Rates

**Figure 3:**
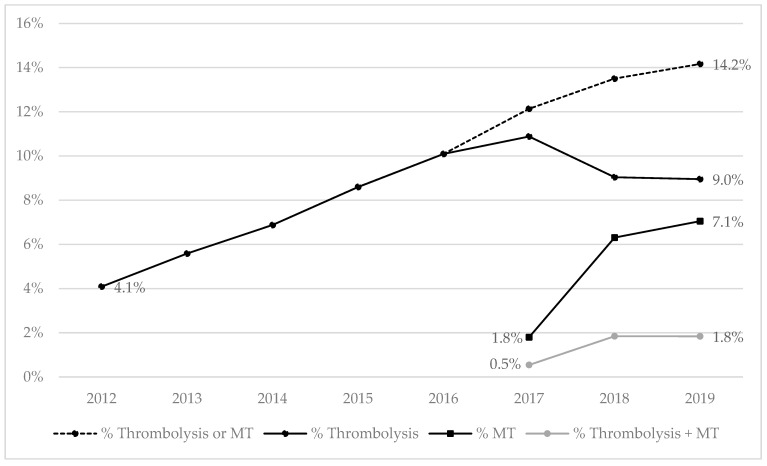
Time trends in the rates of thrombolysis and mechanical thrombectomy (MT) in patients hospitalized for ischemic strokes from 2012 to 2019. Note: Thrombolysis data have only been available since 2012; MT has only been referenced as a medical procedure since August 2017.

In 2019, 60.8% of patients hospitalized for IS were admitted to an acute stroke care unit, representing 40.5% of ICH patients (Table 1). These rates were lower in women compared with men for both IS (56.2% vs. 65.1) and ICH (36.7% vs. 44.0). Among IS patients, the rate of thrombolytic therapy was lower in women (8.6%) than in men (9.2%). Inversely, MT was more frequent in women (7.3%) than in men (6.8%). The use of thrombolytic agents increased over the study period from 4.1% in 2012 (first year available) to 10.9% in 2017 before stabilizing at 10.8% in 2019 (comprised of 9.0% of thrombolysis alone plus 1.8% of patients with both thrombolysis and MT; Figure 3). The use of MT, which began in 2017, increased from 1.8% for this year to 8.9% in 2019 (7.1% receiving MT alone and 1.8% MT plus thrombolysis). 

After adjustment, women were generally less likely to be admitted to an acute stroke care unit (Supplemental Table S1). Nevertheless, non-significant associations between sex and admission to an acute stroke care unit were found in ICH patients aged under 45 years. Patients who were admitted to a geriatric unit or had several comorbidities associated with a Charlson score of 4 or more were less likely to be admitted to an acute stroke care unit.

**Figure 4:**
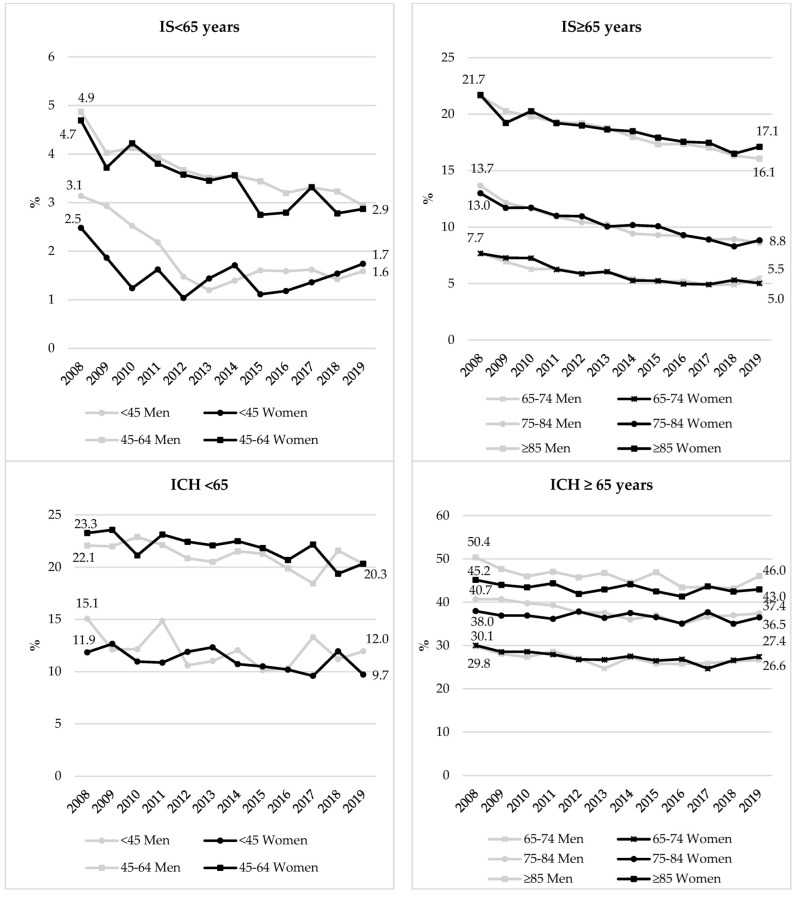
Time trends in the in-hospital case fatality rates (%) for ischemic stroke (IS) and intracerebral hemorrhage (ICH) in France according to sex and age group. Gray: men; black: women.

In 2019, case fatality rates after stroke were higher in women than in men for both IS (10.2% vs. 7.2%) and ICH (33.3% vs. 30.2%) (Table 1; Supplemental Figure S3). After adjustment, women were less likely to die in hospital (OR = 0.95 [95% CI, 0.90–1.00] for IS (*p* = 0.035) and OR = 0.89 [95% CI, 0.83–0.95] for ICH (*p* < 0.0001)) (data not shown). Over the study period, case fatality rates decreased in all age groups for both sexes (Figure 4). The decrease was more pronounced for IS than ICH.

## 4. Discussion

Our study confirmed the increasing trend in the rates of patients hospitalized for IS among people aged under 65 years, particularly those aged 45–64 years and men aged 18–34 and 35–44 years. This trend is accompanied by an increase in the absolute number of men and women hospitalized for IS in these age groups. For the first time, we observed a decrease in the rates of patients hospitalized for ICH since 2013–2014 in all age groups over 50 years for both sexes. Women had a lower chance of being admitted to an acute stroke care unit, as did patients with many comorbidities and those admitted to a geriatric unit.

The increase in the rates of patients hospitalized for IS among people under 65 years has been documented elsewhere for earlier time periods in France and abroad using both administrative databases and population-based stroke registries [3,5,6,10]. In our study, this upward trend was still ongoing in both men and women aged 45–64 years and in men aged 18–34 and 35–44 years. Several hypotheses have been put forward to explain the rise in IS in young and middle-aged men and women. First, there is the higher prevalence of traditional cardiovascular risk factors such as hypertension, hypercholesterolemia, diabetes, obesity, tobacco smoking, and physical inactivity [17,18,19,20,21,22,23,24], which account for more than 80% of the stroke risk in young and middle-aged adults [19]. According to two French nationwide representative population-based studies with clinical examinations in 2006–2007 and 2014–2016, the prevalence of hypertension increased in men aged 18–74 years, whereas it decreased in women of the same age group [25]. The treatment and control of hypertension increased in men but fell in women between the two studies [25]. Based on the same population-based studies, the prevalence of diabetes increased among adults aged 18–74 years [26], particularly the prevalence of undiagnosed diabetes. Simultaneously, a decrease in the prevalence of treated type 2 diabetes was observed in France between 2010 and 2017 among men and women aged 45–64 years [27]. Overall, tobacco smoking remained stable over the study period in France among men and women aged 15–75 years but increased in women aged 50–75 years [28]. Finally, a 21% increase in the prevalence of overweight and obesity was found in women aged 40–54 years in France between 2006 and 2015 according to these two cross-sectional population-based studies [29].

The increased rates of IS in young adults could also be explained by changes in the prevalence of young-specific IS risk factors such as the use of illicit and recreational drugs, migraines, and patent foramen ovale [17]. For instance, the use of cannabis, cocaine, and other drugs sharply increased in adults aged 18–64 years between 2000 and 2017 in France according to the French Monitoring Centre for Drugs and Drug Addiction [30,31]. Similar to our findings, a study based on administrative databases from Cincinnati showed an increase in overall stroke incidence in men aged 20–44 years [32]. Although the higher use of illicit and recreational drugs was observed in both men and women, the prevalence remained much higher in men than in women [33]. More generally, the increase in hospitalized IS in young adults is difficult to evaluate and discuss due to the large proportion of embolic stroke of undetermined source and the potential risk factors in this age category [22,34]. The increase in IS incidence in young adults was hypothesized to be partially related to its better diagnosis due to the more frequent use of brain MRI [35,36]. Changes in the definition of transient ischemic attacks could also partly explain the increase in IS incidence in young adults. However, increases were also found for the incidence of transient ischemic attacks [37].

Among people aged over 65 years, the continuous decline in IS incidence in both men and women was recently reported by the ARIC study [38]. However, we recorded a substantial increase in the absolute number of patients hospitalized for stroke aged over 65 years, particularly in men and the very elderly (≥90 years). These findings should alert health authorities to the increasing and upcoming burden of stroke due to the aging population [14,39,40]. Although a substantial decrease in case fatality rates for IS was observed, the prevalence of IS is predicted to keep rising, leading to the higher prevalence of patients with disabilities and the pressing need to adapt the available health resources to manage these patients, particularly young patients subjected to a long life with disability.

For the first time in France, we recorded the first decrease since 2013–2014 in the rates of patients hospitalized for ICH in both men and women in all age groups over 50 years. This was not the case in our previous work conducted between 2008 and 2014 [8] or in previous data reported by the Dijon Stroke Registry [41]. The massive use of direct oral anticoagulants for the prevention of thromboembolism in patients with atrial fibrillation since 2011–2012 in France could be associated with a decline in the rates of anticoagulant-related ICH. Indeed, direct oral anticoagulants were associated with a lower risk of ICH compared with vitamin K antagonists in patients with atrial fibrillation according to pivotal clinical trials [42], and until 2011–2012, vitamin K antagonists were the only oral anticoagulants available in France. Nevertheless, the absolute number of ICH patients aged over 65 years increased over the study period. This number is expected to further increase with the aging population but also with the increasing number of patients with atrial fibrillation and thus treated with oral anticoagulants [43]. Although case fatality rates decreased for ICH over the study period, only a moderate decrease was observed in 2019, with case fatality rates remaining high.

The sex IRR of patients hospitalized for stroke remained stable over the study period, thus favoring the lower incidence of both IS and ICH in women, with the exception of women aged 18–34 years who are still at a higher risk of IS than men of the same age group. A similar U-shape pattern for the risk of hospitalization for IS in women compared with men was also found in Ontario, Canada [44]. A higher incidence in young women aged 25–44 years was also reported by Leppert et al. in the USA, which is consistent with our findings [45]. Female-specific factors such as migraine, contraceptive medication, and pregnancy might explain this result [17]. Pregnancy and peripartum were found to be associated with a substantially higher risk of IS in previous works [18,44]. The greater occurrence of patent foramen ovale and atrial septum defects was also reported in young women [17,45,46]. On the contrary, the prevalence of traditional cardiovascular risk factors remained higher in young men compared with young women in France [25,26,28,47].

Regarding stroke management in hospital, in 2019 we still observed a lower chance of admission to an acute stroke care unit for women compared with men, as shown in 2014 in France [48]. At that time, the authors explained this result partly by the residual confounding of comorbidities, while they did not take into account stroke severity. Nevertheless, the probability of in-hospital death was lower in women than in men. Patients with a high number of comorbidities or admitted to a geriatric unit were less likely to be admitted to an acute stroke care unit because of the initial selection of patients admitted to these units, especially in the case of an insufficient number of beds. After all adjustments, the addition of one bed to acute stroke care units was associated with a better chance of being admitted, thus highlighting the need to increase the numbers of beds in these units in France. 

Our work has several strengths: the use of nationwide administrative databases allowed the analysis of exhaustive records of hospital data for the entire French population. Furthermore, the high positive predictive value of ICD-10 codes for stroke used in French hospital databases was shown [49]. More than 90% of strokes included in our study had a cerebral imaging even though imaging was not exhaustively reported in hospital discharges. However, unlike population-based stroke registries, this database overlooked stroke patients who were not hospitalized; these patients could account for approximately 4% of all stroke cases. Furthermore, information on the clinical characteristics and vascular history of stroke patients was limited. For instance, no information was available about stroke severity assessed using a specific scale such as the NIHSS score. Finally, we did not include the years 2020 and 2021 in our analyses because the COVID-19 pandemic is known to have challenged the study of time trends of patients hospitalized for stroke, as found in our previous work [50]. 

## 5. Conclusions

Rates of patients hospitalized for IS continued to increase in both men and women aged under 65 years, along with an increase in the absolute number of patients under 75 years. These results highlight the urgent need for an effective stroke prevention program targeting younger and middle-aged adults in France in order to improve the screening, treatment, and control of modifiable vascular risk factors. The encouraging time trends in patients hospitalized for ICH may reflect changes in the use of oral anticoagulants in atrial fibrillation patients, although the absolute number of patients presenting ICH increased over time. Women still had a lower chance of being admitted to an acute stroke care unit in 2019. The reasons for this difference and its impact on long-term survival should be studied in terms of the higher comorbidity profile of women presenting at hospital with stroke, independent of age. Finally, in-hospital case fatality rates decreased in both men and women in all age groups over the study period. Nevertheless, this contributed to an increase in the prevalence of stroke in France, and especially, an increase in the prevalence of people with substantial disabilities. Healthcare resources should be enhanced to manage these patients.

## Figures and Tables

**Table 1:** Number and characteristics of patients hospitalized for ischemic stroke (IS) and intracerebral hemorrhage (ICH) in France in 2019.

	Ischemic Stroke			Intracerebral Hemorrhage		
	All Sex	Men	Women	All Sex	Men	Women
N	97,319	50,503	46,816	18,722	9703	9019
Women, %	48.1	0.0	100.0	48.2	0.0	100.0
Age, mean (SD)	74.2 (14.5)	71.1 (13.8)	77.7 (14.5)	72.3 (16.3)	69.5 (16.3)	75.4 (15.8)
Age, median	77	72	81	76	72	80
Age groups, %						
<18	0.2	0.2	0.2	0.9	1.0	0.7
18–34	1.2	1.0	1.4	2.3	2.7	1.9
35–44	2.4	2.6	2.1	3.2	3.8	2.6
44–54	6.5	8.3	4.5	7.3	8.8	5.6
55–64	12.8	17.3	8.0	12.4	15.3	9.2
65–74	22.0	26.8	16.7	21.4	24.9	17.7
75–84	27.0	26.2	27.9	27.7	26.3	29.1
≥85	28.0	17.7	39.3	24.9	17.2	33.2
Charlson index score (comorbidities), %						
0	34.3	35.4	33.0	39.1	37.2	41.3
1	4.4	4.9	3.9	3.4	3.8	3.0
2	41.2	39.6	42.9	40.0	39.7	40.3
3	6.5	6.8	6.1	5.0	5.8	4.1
≥4	13.7	13.2	14.1	12.5	13.5	11.4
Stroke characteristics, management, and outcomes						
Aphasia, %	44.0	43.1	45.1	33.0	34.3	31.7
Hemiplegia or paraplegia, %	54.1	52.4	55.9	45.7	47.1	44.3
Admission to acute stroke care unit, %	60.8	65.1	56.2	40.5	44.0	36.7
Admission to acute stroke care unit with intensive care, %	52.7	56.6	48.4	34.1	37.6	30.4
Admission to intensive care unit ^†^, %	54.5	58.7	49.9	52.1	56.4	47.4
Admission to geriatric unit, %	8.66	5.8	11.8	8.27	5.9	10.8
Length of stay, mean (SD)	12.3 (13.2)	12.2 (14.1)	12.4 (12.2)	15.2 (20.8)	15.9 (21.4)	14.4 (20.1)
Length of stay, median	9	8	9	10	10	10
Thrombolysis, %	9.0	9.2	8.6	-	-	-
Mechanical thrombectomy, %	7.1	6.8	7.3	-	-	-
In-hospital case fatality rates, %	8.6	7.2	10.2	31.7	30.2	33.3

^†^ including resuscitation unit; SD: standard deviation.

## Data Availability

The data presented in this study are not publicly available.

## Supplementary Materials

The following supporting information can be downloaded at: https://www.mdpi.com/article/10.3390/jcm11061669/s1, Figure S1. Time trends in age-standardized rates of patients hospitalized for ischemic stroke (IS) and intracerebral hemorrhage (ICH) (non-traumatic) between 2008 and 2019 in France according to sex for all ages. Figure S2. Incidence rate ratios (IRR) and 95% confidence interval of patients hospitalized for ischemic stroke (IS) and intracerebral hemorrhage (ICH) between men and women (Women vs. Men) in 2019 according to age group. Figure S3. In-hospital case-fatality rates (%) of patients hospitalized for ischemic stroke (IS) and intracerebral hemorrhage (ICH) by sex and age group in 2019, France. Table S1. Associations with admission to an acute stroke care unit in patients hospitalized for ischemic stroke and intracerebral hemorrhage in 2018–2019 according to age group.
