# Supplementary material for: Patients Hospitalized for Ischemic Stroke and Intracerebral Hemorrhage in France: Time Trends (2008–2019), In-Hospital Outcomes, Age and Sex Differences
Source: J Clin Med. 2022 Mar 17;11(6):1669. doi: 10.3390/jcm11061669 (PMC8949281; doi:10.3390/jcm11061669)
Supplement: Supplementary file 1 [file jcm-11-01669-s001.zip › jcm-1642881-supplementary.pdf]

Supplemental Data

Figure S1. Time trends in age-standardized rates of patients hospitalized for ischemic stroke (IS) and intracerebral hemorrhage (ICH) (non-traumatic) between 2008 and 2019 in France according to sex for **all ages**

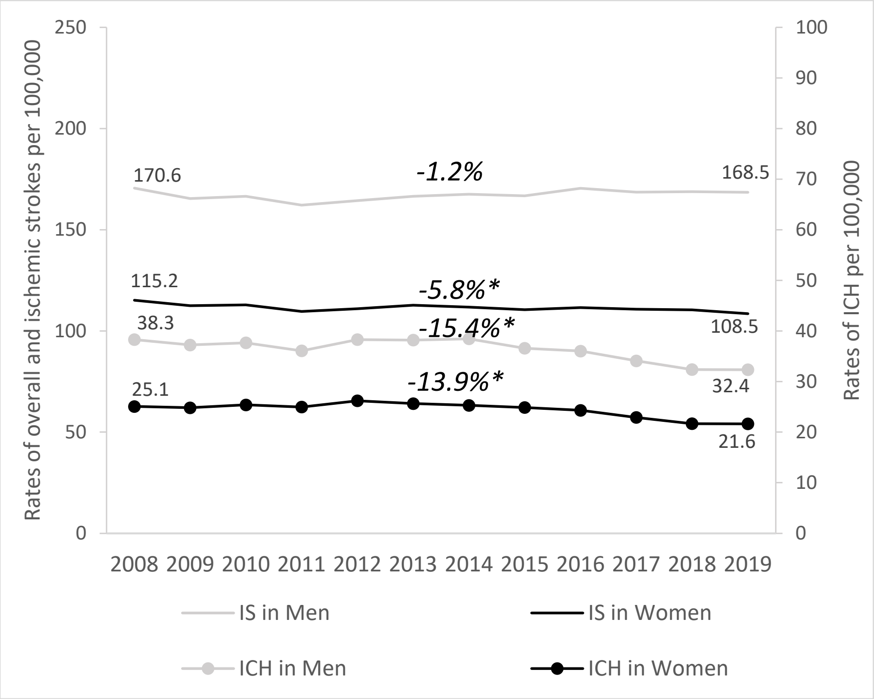

Gray: men; black: women.

**p*-value for tend <0.05

Figure S2. Incidence rate ratios (IRR) and 95% confidence interval of patients hospitalized for ischemic stroke (IS) and intracerebral hemorrhage (ICH) between men and women (Women vs Men) in 2019 according to age group

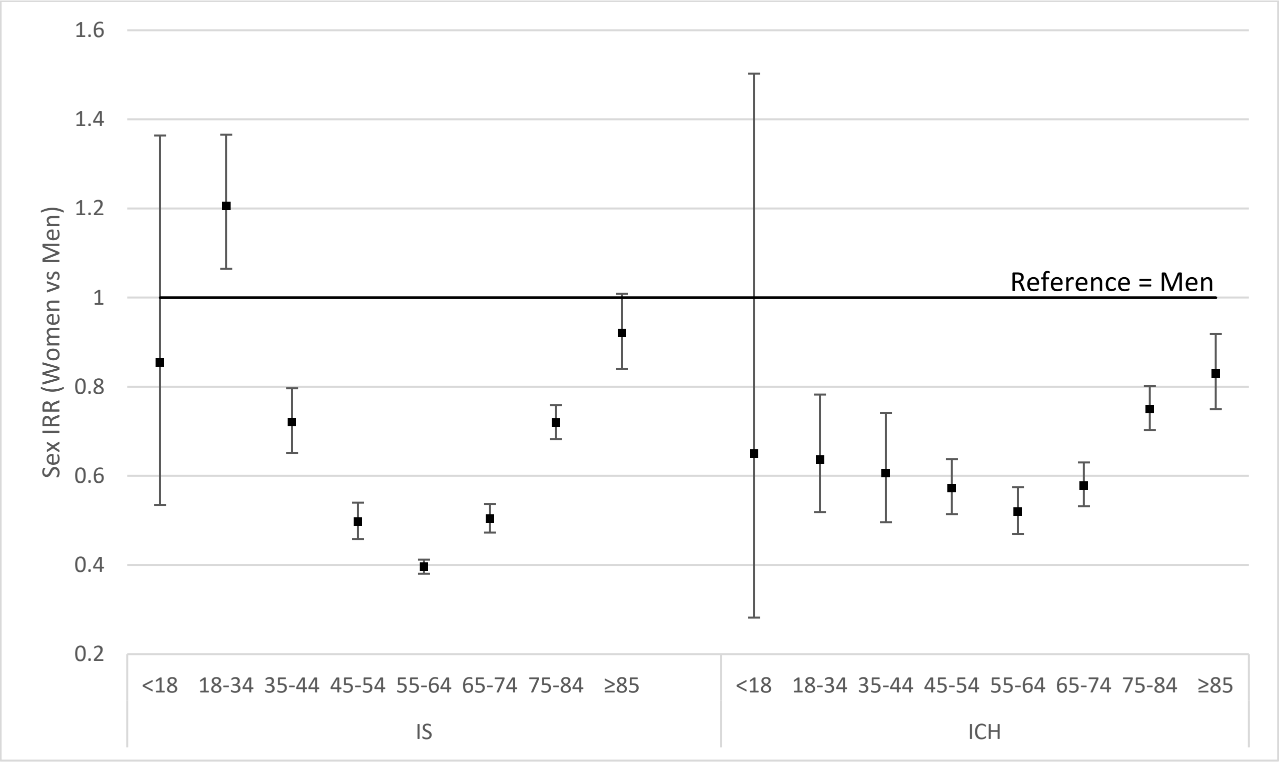

Figure S3. In-hospital case-fatality rates (%) of patients hospitalized for ischemic stroke (IS) and intracerebral hemorrhage (ICH) by sex and age group in 2019, France

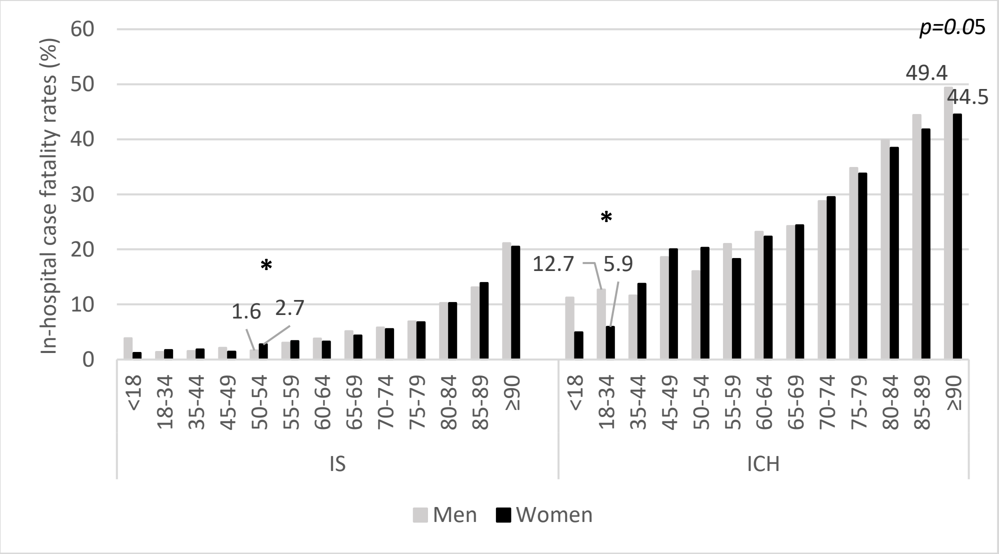

*: *p*-value <0.05

Table S1. Associations with admission to an acute stroke care unit in patients hospitalized for ischemic stroke and intracerebral hemorrhage in 2018-2019 according to age group

	OR†[CI95%]				
	Ischemic stroke				
Effect	<45 yrs	45-64 yrs	65-74 yrs	75-84 yrs	≥85 yrs
Sex (Women vs Men)	**0.87[0.77-0.98]**	**0.82[0.78-0.87]**	**0.88[0.84-0.92]**	**0.86[0.82-0.89]**	**0.87[0.83-0.91]**
Admitted to geriatric unit (yes vs no)	-	-	**0.33[0.28-0.40]**	**0.12[0.11-0.14]**	**0.14[0.13-0.14]**
Charlson score 1 vs 0	0.77[0.54-1.11]	**1.19[1.05-1.34]**	**1.11[1.01-1.23]**	0.99[0.91-1.09]	1.04[0.94-1.16]
Charlson score 2 vs 0	1.22[0.98-1.52]	**1.16[1.07-1.26]**	**1.08[1.01-1.16]**	1.00[0.94-1.06]	1.05[0.99-1.12]
Charlson score 3 vs 0	1.23[0.75-2.00]	1.08[0.94-1.23]	1.04[0.93-1.15]	**0.90[0.82-0.98]**	0.99[0.90-1.09]
Charlson score 4 vs 0	0.69[0.48-1.00]	**0.74[0.67-0.83]**	**0.72[0.66-0.78]**	**0.78[0.73-0.85]**	**0.90[0.83-0.97]**
Aphasia (yes vs no)	**2.88[2.47-3.35]**	**2.48[2.34-2.62]**	**2.39[2.28-2.51]**	**2.50[2.40-2.61]**	**2.64[2.54-2.75]**
Hemiplegia/paraplegia (yes vs no)	**1.83[1.45-2.30]**	**1.73[1.60-1.88]**	**1.83[1.71-1.96]**	**2.21[2.09-2.34]**	**2.33[2.20-2.47]**
Increase of 1 bed in acute stroke care unit*	**1.02[1.01-1.02]**	**1.03[1.02-1.03]**	**1.03[1.03-1.04]**	**1.03[1.03-1.04]**	**1.04[1.04-1.04]**
	Intracerebral hemorrhage				
Effect	<45 yrs	45-64 yrs	65-74 yrs	75-84 yrs	≥85 yrs
Sex (Women vs Men)	0.97[0.79-1.18]	**0.87[0.78-0.97]**	**0.82[0.74-0.91]**	**0.85[0.78-0.94]**	**0.83[0.74-0.92]**
Admitted to geriatric unit (yes vs no)	-	-	**0.49[0.33-0.73]**	**0.20[0.17-0.25]**	**0.17[0.14-0.21]**
Charlson score 1 vs 0	1.67[0.83-3.37]	1.18[0.89-1.58]	1.11[0.85-1.44]	**1.33[1.04-1.71]**	1.20[0.86-1.67]
Charlson score 2 vs 0	0.82[0.56-1.20]	0.88[0.74-1.05]	0.92[0.78-1.09]	1.09[0.94-1.26]	1.06[0.89-1.26]
Charlson score 3 vs 0	1.04[0.53-2.04]	1.05[0.77-1.42]	0.85[0.67-1.09]	1.05[0.83-1.33]	1.23[0.94-1.61]
Charlson score 4 vs 0	**0.49[0.30-0.82]**	**0.48[0.39-0.59]**	**0.54[0.44-0.66]**	**0.71[0.59-0.85]**	0.90[0.72-1.12]
Aphasia (yes vs no)	**2.93[2.28-3.77]**	**3.27[2.91-3.68]**	**3.39[3.04-3.78]**	**3.52[3.18-3.89]**	**3.58[3.18-4.02]**
Hemiplegia/paraplegia (yes vs no)	**2.01[1.37-2.95]**	**3.27[2.76-3.88]**	**3.25[2.77-3.80]**	**3.54[3.08-4.07]**	**3.89[3.30-4.58]**
Increase of 1 bed in acute stroke care unit*	**1.01[1.00-1.02]**	**1.01[1.01-1.02]**	**1.02[1.01-1.02]**	**1.03[1.02-1.03]**	**1.03[1.03-1.04]**

OR: odds ratio; CI: confidence interval; yrs: years; bold: *p*-value<0.05.

†models adjusted for age, sex, admission to a geriatric unit during the hospital stay, Charlson index score (categorized in five class), presence of aphasia, hemiplegia, or paraplegia, and the number of beds available in acute stroke care units in the department where the patient lives.

*in the patient's department of residency
